# Identifying Distinguishable Clinical Profiles Between Single Suicide Attempters and Re-Attempters

**DOI:** 10.3389/fpsyt.2021.754402

**Published:** 2021-09-27

**Authors:** Marlehn Lübbert, Lydia Bahlmann, Sebastian Josfeld, Jessica Bürger, Alexandra Schulz, Karl-Jürgen Bär, Udo Polzer, Martin Walter, Ulrich W. Kastner, Thomas Sobanski, Gerd Wagner

**Affiliations:** ^1^Department of Psychiatry and Psychotherapy, Jena University Hospital, Jena, Germany; ^2^Department of Psychiatry, Psychotherapy, and Psychosomatic Medicine, Thüringen-Kliniken Georgius Agricola GmbH, Saalfeld, Germany; ^3^Department of Gerontopsychiatry and Psychosomatics, Jena University Hospital, Jena, Germany; ^4^Clinics for Psychiatry, Psychotherapy and Addition Disorders, Asklepios Fachklinikum Stadtroda, Stadtroda, Germany; ^5^Department of Psychiatry and Psychotherapy, Helios Fachkliniken Hildburghausen, Hildburghausen, Germany

**Keywords:** suicidal behavior disorder, suicide re-attempt, personality, clinical profiles, suicidal ideation

## Abstract

More than 800,000 individuals die from suicide each year in the world, which has a devastating impact on families and society. Ten to twenty times more attempt suicide. Previous studies showed that suicide attempters represent a heterogeneous group regarding demographic characteristics, individual characteristics of a suicidal attempt, and the assumed clinical factors, e.g., hopelessness or impulsivity, thus differently contributing to the likelihood of suicidal behavior. Therefore, in the present study, we aim to give a comprehensive clinical description of patients with repeated suicide attempts compared to single attempters. We explored putative differences between groups in clinical variables and personality traits, sociodemographic information, and specific suicide attempt-related information. A sample of patients with a recent suicide attempt (*n* = 252), defined according to DSM-5 criteria for a suicidal behavior disorder (SBD), was recruited in four psychiatric hospitals in Thuringia, Germany. We used a structured clinical interview to assess the psychiatric diagnosis, sociodemographic data, and to collect information regarding the characteristics of the suicide attempt. Several clinical questionnaires were used to measure the suicide intent and suicidal ideations, depression severity, hopelessness, impulsivity, aggression, anger expression, and the presence of childhood trauma. Univariate and multivariate statistical methods were applied to evaluate the postulated risk factors and, to distinguish groups based on these measures. The performed statistical analyses indicated that suicide attempters represent a relatively heterogeneous group, nevertheless associated with specific clinical profiles. We demonstrated that the re-attempters had more severe psychopathology with significantly higher levels of self-reported depression, suicidal ideation as well as hopelessness. Furthermore, re-attempters had more often first-degree relatives with suicidal behavior and emotional abuse during childhood. They also exhibited a higher degree of specific personality traits, i.e., more “urgency” as a reaction to negative emotions, higher excitability, higher self-aggressiveness, and trait anger. The multivariate discriminant analysis significantly discriminated the re-attempters from single attempters by higher levels of self-aggressiveness and suicidal ideation. The findings might contribute to a better understanding of the complex mechanisms leading to suicidal behavior, which might improve the early identification and specific treatment of subjects at risk for repeated suicidal behavior.

## Introduction

More than 800,000 individuals die from suicide each year in the world; thus every 40 s a person commits suicide (1). This has a devastating impact on families and society. The psychological, social, and financial impacts on the family and community are immeasurable.

Despite an exponential increase in the number of publications in the field of suicidology in recent years (2), prediction of future suicidal behavior (SB) and treatment of individuals after a suicide attempt (SA) remain a significant challenge. For instance, the suicide rate in the first 3 months after psychiatric hospital discharge is 100 times higher than the global suicide rate, particularly among patients admitted with SB, indicating the strong need for better prediction and treatment (3).

Many previous studies focused on specific clinical and personality factors that might enhance the likelihood of suicidal behavior. For instance, these studies found an association between suicide attempts and the presence of psychiatric disorders (4–6), greater hopelessness (6, 7), higher level of impulsivity and aggression (8, 9) and the presence of childhood trauma (10–12).

However, a recent meta-analysis markedly revealed that 50 years of research had improved our knowledge about SB and suicidal ideations (SI). However, it has had a limited impact on increasing our knowledge about suicide/SA prediction (13). Most of the assumed clinical risk factors for SB, as was long believed, are predictive for the emergence of suicidal ideations (13). For instance, the predictive role of mental disorders, like Major Depressive Disorder (MDD), has been considered as one of the most significant risk factors for a long time. However, this association primarily exists because mental disorders facilitate the development of SI but do not distinguish suicide attempters from ideators (14–16). Thus, the only clinically relevant predictor for future SB to date is the history of previous suicide attempts (13, 17–19).

Several reasons partially explain this slow progress. One of the reasons can be found in the complex and multifactorial pathophysiological mechanisms leading to SB that make its prediction and prevention still challenging (20–22). For instance, the exclusive presence of specific psychosocial factors, like the experience of acute stressful conditions (e.g., interpersonal conflicts), did not explain the development of SB or still suicidal ideation. Instead, there is strong consensus on the notion that a combination of several risk factors is required to exhibit SI and additional factors to transit from suicidal ideation to acts (19). To address these complex multifactorial mechanisms, stress-diathesis-models e.g., J. John Mann and Mina M. Rizk (23) and ideation-to-action models, e.g. Joiner (24) and Van Orden et al. (25) were proposed.

Furthermore, the different and often idiosyncratic definitions of SB and self-harm, used in previous studies and thus leading to several phenotypes along the suicide continuum may have contributed to a huge variability regarding risk factors associated with SB and make it difficult to summarize, integrate, and compare previous findings (26, 27). Only recently, the Diagnostic and Statistical Manual of Mental Disorders 5 (DSM-5) proposed criteria for “suicidal behavior disorder” (SBD) to establish a common language for researchers and clinicians as well as to set the basis for improved identification and definition (28).

In addition, previous studies, as well as clinical experience, clearly recognize that suicide attempters cannot be seen as a homogeneous group because of the significant variability in clinical factors, sociodemographic aspects, personality profiles, as well as regarding specific characteristics of the suicide attempt (29).

Therefore, a better understanding of SB and thus more knowledge about specific markers at the individual level are urgently needed to improve our ability to predict future SA and suicide as well as to implement effective suicide prevention and intervention strategies.

Thus, we aimed in our study to explore different patterns of risk factors based on previous findings and theories in an integrative way, specifically focusing on the group of patients with the highest risk for a future suicide attempt or suicide, i.e., patients with past suicide attempt(s).

Previous studies showed significant differences in sociodemographic and psychopathological profiles between single suicide attempters vs. re-attempters. Regarding sociodemographic factors, the repeated attempters were shown to have younger age, being often females (30), unemployed and living alone as well as having lower educational status (31). In addition, it was also demonstrated that re-attempters had in general more severe psychopathology that included higher levels in affective, anxiety, psychotic, PTSD symptoms and higher frequency of comorbid, alcohol, and substance abuse disorders (31, 32).

Other clinical factors related to re-attempters compared to single-attempters included family history of suicidal behavior, presence of childhood trauma and/or emotional abuse, higher scores in hopelessness and motor impulsivity, lifetime history of aggressive behavior, poorer interpersonal functioning (e.g., deficits in conflict resolution skills) and a greater number of stressful life events (33, 34).

To sum up, suicide attempters are recognized as a high-risk group, underscoring the importance of identifying specific characteristics, e.g., clinical or personality markers, that might improve the accurate detection of future suicide re-attempters. On this basis, prevention strategies like targeted psychotherapeutic or psychosocial interventions could be optimized. Therefore, in the present study, we aim to give a comprehensive clinical description of patients with repeated suicide attempts compared to single attempters. To achieve this goal, we explored putative differences between groups in clinical variables and personality traits, sociodemographic information, and specific suicide attempt-related information. Based on previous studies, we expected to find significant differences in these variables between the high-risk subgroups of single- vs. re-attempters.

## Materials and Methods

### Subjects

A sample of adult inpatients (*n* = 252) with a current suicide attempt (SA) was recruited from 2018 to 2020 in four different cooperating sites in Thuringia, Germany (Departments of Psychiatry and Psychotherapy of the University Hospital Jena, of the Thüringen-Kliniken “Georgius Agricola” Saalfeld, of the Asklepios Fachklinikum Stadtroda and the Helios Fachkliniken Hildburghausen), as part of an ongoing suicide prevention project (“Network for suicide prevention in Thuringia”), founded by the federal ministry of health (BMG). We only included subjects who fulfilled the DSM-5 (35) criteria for the current suicidal behavior disorder (SBD). In the DSM-5, “suicide attempt” is explicitly defined as “a self-initiated sequence of behaviors by an individual who, at the time of initiation, expected that the set of actions would lead to his or her own death.” Since this definition strongly emphasizes the intent to die, we have systematically assessed it using the Suicide Intent Scale, SIS (36) in our study. The current diagnosis of SBD was thus clearly differentiated from the “Non-suicidal Self-Injury” (NSSV), another condition for further study in DSM-5. We also used a more conservative time criterion than setting down in DSM-5, i.e., not more than 12 months since the recent attempt. According to DSM-5, exclusion criteria were acute psychosis, acute intoxication, withdrawal symptoms, diagnosed intelligence impairment, language barriers, lack of insight, and dementia diseases. Based on these criteria, we excluded 40 participants so that the final sample included *n* = 212 participants. Table 1 presents further information about sample characteristics. The local ethics committees of the Friedrich-Schiller University, Jena and of State Chamber of Physicians of Thuringia, Germany approved the study. Informed written consent was obtained from all participants before their participation. Patients were contacted and interviewed by a trained psychologist with a Master degree (M.L., S.J., L.B., and A.S.). Subsequently, questionnaires were explained and given to the patients to fill out in the following days.

**Table 1:** Sociodemographic characteristics of single- and re-attempters' groups.

**Characteristics**	**Single attempters**	**Re-attempters**	**Test statistic**	***df***	***p***
	(***n*** **= 99)**	(***n*** **= 113)**			
Gender (% male)	64 (64.6%)	48 (42.5%)	χ^2^ = 10.018	1	0.002**^b^
Age (years) (Mean ± SD)	49.16 ± 19.3	39.2 ± 17.5	*t* = 3.94	210	<0.0001**^c^
Suicide intent scale (Mean ± SD)	12.13 ± 5.18	12.45 ± 4.21	*t* = −0.49	205	0.62^c^
Number of past suicide attempts (Mean ± SD)	–	2.79 ± 3.0			
Time between the last suicide attempt and the interview in months (Mean ± SD)	0.42 ± 1.29	0.74 ± 1.52	*t* = −1.63	210	0.104^c^
**Education**					
Special needs school	1 (1.0%)	2 (1.8%)			
8 years school	18 (18.2%)	28 (24.8%)			
10 years school	54 (54.5%)	54 (47.8%)			
12 years school	11 (11.1%)	18 (15.9%)			
University/college	12 (12.1%)	10 (8.8%)			
Family status			χ^2^ = 16.539	4	0.002**^b^
Unmarried	34 (34.3%)	69 (61.1%)			
Divorced	13 (13.1%)	8 (7.1%)			
Widowed	9 (9.1%)	5 (4.4%)			
Married, but living apart	8 (8.1%)	10 (8.8%)			
Married	34 (34.3%)	21 (18.6%)			
Employed (%)^a^	48 (48.5%)	55 (48.7%)	χ^2^ = 0.048	1	0.827^b^
Living alone (%)	36 (36.4%)	43 (38.1%)	χ^2^ = 0.161	1	0.688^b^
Number of children (Mean ± SD)	1.41 ± 1.32	0.88 ± 1.15	*Z* = −3.35		0.001**^d^
1st degree relatives with suicidal behavior (%)	9 (9.1%)	23 (20.4%)	χ^2^ = 5.223	1	0.022*^b^
Means of suicide attempt (% non-violent)	67 (67.7%)	72 (63.7%)	χ^2^ = 0.367	1	0.565^b^
Previous psychiatric/ psychotherapeutic treatment (%)	46 (46.5%)	100 (88.5%)	χ^2^ = 142.521	1	<0.0001**^b^
**Psychiatric disorder (DSM-IV)**					
*MDD*	65 (65.7%)	65 (57.5%)			
*BPD*	3 (3.0%)	19 (16.8%)			
*Substance abuse*	7 (7.1%)	10 (8.8%)			
*BD*	6 (6.1%)	8 (7.1%)			
*AD*	11 (11.1%)	2 (1.8%)			
*ASD/PTSD*	4 (4.0%)	2 (1.8%)			
*Phobic disorder*	–	3 (2.7%)			
*OCD*	1 (1.0%)	1 (0.9%)			
*Other PD*	1 (1.0%)	1 (0.9%)			
*Autism*	–	1 (0.9%)			
*Somatoform disorder*	1 (1.0%)	–			
*Pathological gambling*	–	1 (0.9%)			
**Psychotropic medication**					
*SSRI*	25 (25.3%)	30 (26.5%)			
*SSNRI*	21 (21.2%)	27 (23.9%)			
*SNDI*	–	4 (3.5%)			
*SNRI*	–	2 (1.8%)			
*SARI*	–	2 (1.8%)			
*NaSSA*	35 (35.4%)	29 (25.7%)			
*AAP*	26 (26.3%)	50 (44.2%)			
*TAP*	4 (4.0%)	13 (11.5%)			
*TCA*	2 (2.0%)	11 (9.7%)			
*BZD*	13 (13.1%)	10 (8.8%)			
*LiS*	4 (4.0%)	8 (7.1%)			
*AED*	3 (3.0%)	12 (10.6%)			
*APD*	–	2 (1.8%)			
*DA*	1 (1.0%)	–			
*MA*	1 (1.0%)	–			
**Motives for suicide attempt**					
*Interpersonal conflicts*	59 (59.6%)	71 (62.8%)	χ^2^ = 0.233	1	0.629^b^
*Acute stressful events*	30 (30.3%)	50 (44.2%)	χ^2^ = 4.367	1	0.037*^b^
*Persistent stressful circumstances and experience of overstrain*	16 (16.2%)	18 (15.9%)	χ^2^ = 0.002	1	0.963^b^
*Expectation of severe somatic or mental disorder*	14 (14.1%)	2 (1.8%)	χ^2^ = 11.575	1	0.001**^b^
*Psychiatric symptoms*	16 (16.2%)	31 (27.4%)	χ^2^ = 3.886	1	0.049*^b^
*Somatic symptoms*	17 (17.2%)	9 (8.0%)	χ^2^ = 4.157	1	0.041*^b^
*Burdensomeness*	20 (20.2%)	33 (29.2%)	χ^2^ = 2.280	1	0.131^b^
*Disconnectedness*	18 (18.2%)	32 (28.3%)	χ^2^ = 3.009	1	0.083^b^
*Hopelessness*	57 (57.6%)	69 (61.1%)	χ^2^ = 0.266	1	0.606^b^
*Fear of the future*	14 (14.1%)	9 (8.0%)	χ^2^ = 2.082	1	0.149^b^

* *p < 0.05*,

** *p <0.01*.

a *Being employed include fulltime or part-time employment, guarded employed, volunteering work, federal volunteer service and being in training or retraining, students, and pupils*.

b *χ^2^-test*.

c *T-test with (assumed) equal variances*.

d *Mann-Whitney-U-Test*.

*MDD, major depressive disorder; BPD, borderline personality disorder; SUD, substance use disorder; BD, bipolar disorder; AD, adjustment disorder; ASD/PTSD, acute stress disorder/ post-traumatic stress disorder; PD, phobic disorder; OCD, obsessive-compulsive disorder; PD, personality disorder; ASD, autism spectrum disorder; SoD, somatoform disorder; PG, pathological gambling; SSRI, selective serotonin reuptake inhibitors; SSNRI, selective serotonin-norepinephrine reuptake inhibitors; SNDI, serotonin and norepinephrine disinhibitors; SNRI, serotonin and norepinephrine reuptake inhibitors; SARI, serotonin antagonist and reuptake inhibitors; NaSSA, noradrenergic and specific serotonergic antidepressant; AAP, atypical antipsychotics; TAP, typical antipsychotics; TCA, tricyclic antipsychotics; BZD, benzodiazepine; LiS, lithium salt; AED, antiepileptic drugs; APD, antiparkinson drugs; DA, dopamine-agonist; MA, melatonin-agonist*.

### Clinical Assessment Tools

The data collection included structured interviews and a comprehensive battery of questionnaires to measure clinical symptoms and personality traits. The presence of psychiatric diseases was assessed by trained psychologists using the Mini International Neuropsychiatric Interview, M.I.N.I. (37), a short structured diagnostic interview for DSM-IV Axis I disorders. We also collected systematic information about the sociodemographic characteristics, the number of suicide attempts, familial history of suicidal behavior among the first-degree degree relatives, number of past psychiatric/ psychotherapeutic treatments, medication status, and the circumstances and trigger of the recent suicide attempt. Suicidal ideations during the past week were assessed using the German version of the Beck Scale for Suicide Ideation, BSS (38). Depressive symptoms were evaluated by the Montgomery-Åsberg Depression Rating Scale, MADRS (39), and *via* self-report using the Revised Becks Depression Inventory, BDI-2 (40). Hopelessness was measured with the revised version of the validated German Hopelessness Scale (41), based on Beck's cognitive theory of depression (42), and which explores pessimism concerning the future. Impulsivity was assessed by a German version of the Impulsive Behavior Scale, UPPS (43), exploring four dimensions of impulsivity: lack of premeditation, urgency, sensation seeking, and lack of perseverance. To evaluate aggressive traits, two questionnaires were used. The first one, a short version of the validated and widely used German Questionnaire for Assessing Factors of Aggression, K-FAF (44), was applied to measure readiness for aggressive behavior, including spontaneous aggressiveness, reactive aggressiveness, excitability, self-aggressiveness, and aggression inhibition. The second questionnaire, the State-Trait Aggression Inventory, STAXI-2 (45), was used to explore the intensity of anger as an emotional state and the disposition to experience angry feelings as a personality trait. Finally, we assessed childhood trauma using the Childhood Trauma Scale, CTQ (46), exploring physical abuse, sexual abuse, emotional abuse, physical neglect, and emotional neglect during infancy.

### Statistical Analysis

We used SPSS Version 21.0 (https://www.ibm.com/de-de/analytics/spss-statistics-software) for the statistical analyses. To investigate differences in categorical variables, i.e., in sociodemographic factors, motives, and triggers of the current suicide attempt, the non-parametric χ^2^-test was used. In addition, Mann-Whitney U-test was utilized for measures with the non-Gaussian distribution. To explore the assumed differences in clinical risk factors (e.g., suicidal ideation, hopelessness, depression) Student's *t*-tests for continuous variables were calculated based on the data of clinical questionnaires.

The whole sample of patients was compared using *t*-tests (https://www.medcalc.org/calc/comparison_of_means.php) regarding personality traits to the norm samples as specified in the questionnaire's related manuals, i.e., K-FAF (47) and STAXI-2 (45) or in the corresponding publication, i.e., UPPS (43). A Bonferroni correction was applied for controlling Type I error due to multiple comparisons. In addition, we used a logistic regression to explain single attempts vs. re-attempts by the binarized motives/triggers of the current suicide attempt and sociodemographic factors. We further used the discriminant analysis, as a multivariate model, to differentiate the single- and the re-attempters based on the assessed clinical and personality questionnaires. This method was used to find a linear combination of variables that separates both studied groups of suicide attempters. The resulting combination can be used as a linear classifier.

## Results

### Single- vs. Re-attempters

#### Sociodemographic Data

We found significant differences in the sociodemographic characteristics between single attempters and re-attempters. The latter patients were significantly younger, included more females, were often unmarried, had significantly fewer children and had significantly more first-degree relatives with a familial history of suicidal behavior (see Table 1). Furthermore, the re-attempters were significantly more often in psychiatric/psychotherapeutic treatments before the recent suicide attempt compared to the single attempters.

#### Clinical Data and Personality Traits

We did not observe any significant differences between both groups regarding the intent to die as assessed by SIS (see Table 1). However, several significant differences were found in clinical questionnaires, as depicted in Table 2. The re-attempters had significantly higher scores in suicidal ideation (BSS) as well as in self-reported depression severity (BDI-2) and hopelessness (H-R-scale). Interestingly, no significant difference could be found in the clinician-based depression severity (MADRS).

**Table 2:** Clinical characteristics and personality traits of single- and re-attempters' groups.

	**Single attempters**	**Re-attempters**	**Test statistic**	***df***	***p***
	(***n*** **= 99)**	(***n*** **= 113)**			
**General psychopathology**					
Montgomery-Åsberg Depression Rating Scale (MADRS)	19.26 ± 10.94	22.39 ± 10.02	*t* = −2.04	183	0.043*
Beck Depression Inventory (BDI 2)	18.42 ± 13.49	27.80 ± 12.87	*t* = −4.30	144	<0.0001**^b^
Hopelessness Scale (H-R-Skala)	62.11 ± 20.78	76.92 ± 20.15	*t* = −4.56	157	<0.0001**^b^
Beck Scale for Suicide Ideation (BSS)	3.30 ± 7.05	10.74 ± 10.58	*t* = −5.1	153	<0.0001**^b^
**Aggression, impulsivity, and childhood trauma**					
***Impulsive Behavior Scale (UPPS)***					
Urgency	30.10 ± 5.68	32.83 ± 6.65	*t* = −2.71	151	0.007**
Premeditation	21.85 ± 4.92	23.69 ± 5.39	*t* = −2.19	150	0.030*
Perseverance	20.23 ± 5.27	22.26 ± 5.68	*t* = −2.28	150	0.024*
Sensation seeking	26.57 ± 7.61	27.57 ± 8.01	*t* = −0.79	151	0.432
***Short Questionnaire for Assessing Factors of Aggression (K-FAF)***					
Spontaneous aggressiveness	10.66 ± 8.75	13.82 ± 10.14	*t* = −1.89	129	0.062
Reactive aggressiveness	21.88 ± 9.52	25.11 ± 11.62	*t* = −1.71	128	0.090
Excitability	17.71 ± 10.39	23.85 ± 11.94	*t* = −3.10	129	0.002**^b^
Self-aggressiveness	22.92 ± 10.12	31.53 ± 9.42	*t* = −5.04	129	<0.0001**^b^
Aggression inhibition	19.98 ± 6.09	18.85 ± 6.31	*t* = −1.04	129	0.300
Total score^a^	50.25 ± 24.63	62.68 ± 29.78	*t* = −2.56	128	0.012*
**State-Trait Anger Expression Inventory (STAXI-2)**					
Trait Anger	20.18 ± 6.12	24.04 ± 7.00	*t* = −3.33	129	0.001**^b^
Expression out	12.90 ± 4.92	14.51 ± 5.03	*t* = −1.84	129	0.068
Expression in	20.63 ± 5.49	22.31 ± 5.90	*t* = −1.73	129	0.087
Anger control	30.50 ± 6.62	28.31 ± 6.68	*t* = 1.88	129	0.063
**Childhood Trauma Questionnaire (CTQ)**					
Emotional abuse	9.12 ± 5.13	12.10 ± 5.13	*t* = −3.38	154	0.001**^b^
Physical abuse	6.86 ± 3.74	8.42 ± 4.76	*t* = −2.26	155	0.025*
Sexual abuse	6.14 ± 4.22	7.86 ± 5.24	*t* = −1.73	152	0.027*
Physical neglect	8.47 ± 4.98	9.79 ± 5.43	*t* = −1.31	156	0.038*
Emotional neglect	11.0 ± 3.79	12.58 ± 4.08	*t* = −1.58	155	0.061

* *p < 0.05*,

** *p < 0.01*.

a *Comprise the subscales spontaneous aggression, reactive aggression, and excitability*.

b *Remains significant after a Bonferroni correction*.

Nearly 50% of suicide attempters reported emotional abuse in the whole sample, and nearly 60% reported emotional and physical neglect during their childhood. As shown in Table 2, the re-attempters had significantly higher scores only for the emotional abuse scale in the CTQ compared to single attempters.

**Figure 1:**
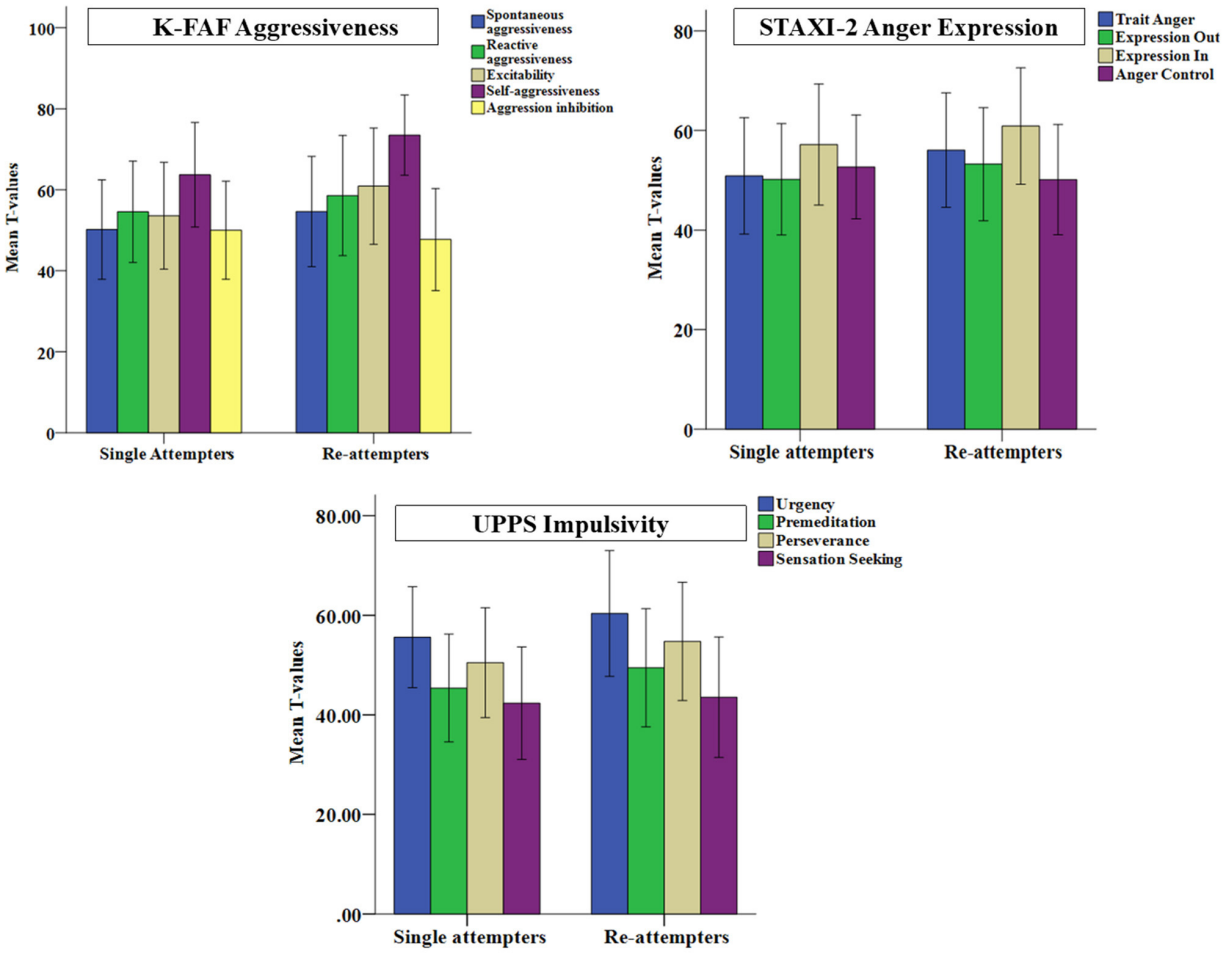
Standardized values (*T*-scores) computed from the corresponding norm samples are separately depicted for the single attempters and the re-attempters for the subscales of the German questionnaire to measure aggressiveness factors (K-FAF), of the State-Trait Anger Expression Inventory (STAXI-2), and of the Impulsive Behavior Scale (UPPS).

Regarding the investigated personality traits, we observed in the whole sample of suicide attempters significantly higher impulsivity (UPPS) scores for the trait “urgency” (*M* = 31.5, *SD* = 6.3) compared to the reference sample (43) and significantly lower scores for “sensation seeking” (*M* = 27.1, *SD* = 7.8) at the Bonferroni adjusted alpha level of 0.05, but not for two other impulsivity traits. Furthermore, compared to single attempters, the re-attempters exhibited higher scores in three of four UPPS subscales. However, the *p*-values did not survive the Bonferroni correction (see Table 2 and Figure 1).

In addition, compared to the norm sample (47) significantly elevated aggression scores (*p* < 0.05, Bonferroni corrected) were found in the whole sample for the total aggression score (*M* = 57.0, *SD* = 28.2) and for all subscales of the K-FAF (“spontaneous aggressiveness”: *M* = 12.4, *SD* = 9.6; “reactive aggressiveness”: *M* = 23.7, *SD* = 10.8; “excitability”: *M* = 21.1, *SD* = 11.6; self-aggressiveness: *M* = 27.7, *SD* = 10.6) except “aggression inhibition.” Compared to single attempters, the re-attempters scored significantly higher in K-FAF subscales “excitability,” “self- aggressiveness” (see Table 2 and Figure 1).

Regarding the State-Trait Aggression Inventory (STAXI-2), the whole sample of patients showed significantly higher scores in all subscales (“trait anger”: *M* = 22.3, *SD* = 6.9; “expression out”: *M* = 13.8, *SD* = 5.0; “expression in”: *M* = 21.5, *SD* = 5.6), except “anger control” (*p* < 0.05, Bonferroni corrected). But the re-attempters scored significantly higher than single attempters only in “trait anger” (see Table 2 and Figure 1).

### Triggering Factors of the Current Suicide Attempt

Interpersonal conflicts (61%) and other acute stressful events (38%), as well as hopelessness (59%), were reported most frequently as motives and triggering factors of the current suicide attempt. Around 25% of patients also indicated two interpersonal constructs of Joiner's ideation-to-action model (25), i.e., perceived burdensomeness and disconnectedness, as central motives for their suicide attempt. However, comparing single attempters vs. re-attempters, no significant group differences were found regarding these triggering factors. Furthermore, the re-attempters indicated significantly more often acute stressful events and the presence of psychiatric symptoms. In contrast, single attempters indicated significantly more often the expectation of a severe somatic and/or mental disorder and the presence of somatic symptoms as triggering factors for the last suicide attempt (see Table 1). The logistic regression with sociodemographic and motives/triggering factors conferred the results of single χ^2^-tests revealing, that suicide re-attempts were significantly predicted by acute stressful events (β = 0.67, *p* = 0.039), younger age (β = −0.03, *p* = 0.002) and female gender (β = −0.8, *p* = 0.009), and single-attempts by the expectation of a severe somatic and mental disorder (β = −2.270, *p* = 0.008).

### Most Discriminating Clinical and Personality Factors

**Figure 2:**
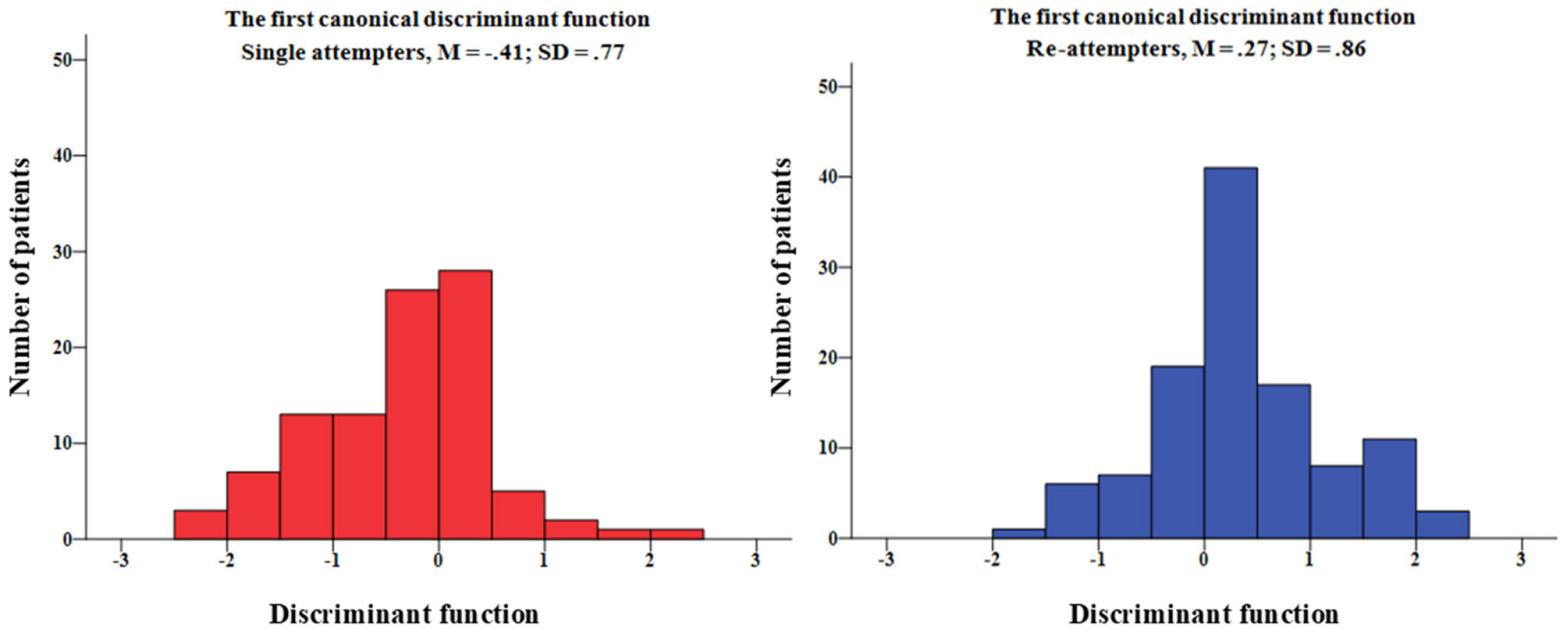
Histograms of the first canonical discriminant function scores are separately depicted for single- and re-attempters. The standardized canonical discriminant function coefficients were for K-FAF self-aggression 0.65 and for BSS suicide ideation 0.55.

Finally, using step-wise discriminant analysis, the variables, self-aggressiveness and suicidal ideation, were identified as the two factors, which significantly discriminated between the single- and the re-attempters groups (in the first step with “K-FAF self-aggression” W⋌ = 0.83, *F* = 18.75, and *p* < 0.0001, in the second step with “BSS total score” W⋌ = 0.78, *F* = 12.49, and *p* < 0.0001). Wilks' Lambda test was used to indicate which variable significantly contributes to the discriminant function. The closer Wilks' lambda is to 0, the more the variable contributes to the discriminant function. The standardized canonical discriminant function coefficients were for self-aggression 0.65 and for suicide ideation 0.55. According to the structure matrix, the correlations of K-FAF subscale “self-aggressiveness” and BSS total score with the discriminant function were 0.86 and 0.80, respectively. We also assessed how well the discriminant function works. 67.0% of the cases were correctly classified in the present study (see Figure 2).

### Reaction Toward Survival After a Suicide Attempt

Using Mann–Whitney *U*-test, we compared single attempters and the re-attempters regarding their reaction toward survival after a suicide attempt at the time of the interview measured on a 3-point scale (negative = −1, indifferent = 0, and positive = 1). Patients in both groups mostly showed a positive reaction toward their survival. However, single attempters (*M* = 0.69, *SD* = 0.59) reported significantly more often positive reactions (*Z* = −3.2, *p* = 0.001) than the re-attempters (*M* = 0.39, *SD* = 0.74).

## Discussion

The large diversity in risk factors and theoretical models does not significantly contribute to a better-than-chance prediction of future suicidal behavior (13, 48). As a result, suicide risk assessment remains vague, leading to uncertainties in therapists, nurses, and, thus, in some cases to inappropriate treatment strategies with negative consequences for patients. In addition, because of the heterogeneity of suicide attempters, differing clinical profiles should be considered in risk assessment and subsequent treatment.

Thus, we focused on the differences between single- and re-attempters in sociodemographic, clinical, and personality measures in the present study. We investigated a sample of suicide attempters using a comprehensive battery of structured interview, clinical, and personality questionnaires. In agreement with previous studies, suicide re-attempters differed significantly from single attempters in several sociodemographic characteristics. They were significantly younger, often female, unmarried, had fewer children and had more first-degree relatives with suicidal behavior than single attempters. They also reported more previous psychiatric/psychotherapeutic treatments, which, given a more severe psychopathological profile, is reasonable (49). Thus, present findings confirmed the previously assumed risk factors for a suicide re-attempt [e.g., (31, 32)]. Furthermore, we extended these previous findings by providing additional information about specific facets of impulsivity, aggressiveness, anger processing, and clinical characteristics.

We demonstrated that the suicide re-attempters had more severe psychopathology with significantly higher levels of self-reported depression, suicidal ideation as well as hopelessness. Furthermore, emotional abuse during childhood and specific personality traits, i.e., more urgency as a reaction to negative emotions, higher excitability, more self-aggressiveness, and higher scores in trait anger, were found in the suicide re-attempters. Finally, the discriminant analysis discriminated the re-attempters from single attempters by higher levels of self-aggressiveness and suicidal ideation in the re-attempters group. These are new promising findings with a high potential for optimizing risk assessment and treatment.

Interestingly, suicidal ideation remained high after the suicide attempt, especially in the re-attempters group. This finding seems surprising, as a cathartic relieving effect after the suicidal act was observed in some clinical patients. Matsuishi et al. (50) investigated in a study with 88 suicide attempters the severity of suicidal ideation immediately before and after the suicide attempt. They found significantly decreased suicidal ideation after the suicide attempt that varied among different ages (only significant decrease in patients under 60 years old) and psychiatric disorders, conferring the catharsis-hypothesis that suicide appears to be an ultimate form of catharsis (51, 52). In the present study suicidal ideation remained relatively high in the re-attempters compared to single attempters indicating a less cathartic effect. Accordingly, Joiner and Rudd (53) found out that re-attempters experienced a longer duration of suicidal crises in response to negative life events. The presence of higher suicidal ideation and lower relieving effect of a suicide attempt observed in the present study points into the direction of a prolonged suicidal crisis in the re-attempters than single attempters.

The other most discriminating factor was the degree of self-aggressiveness that was much higher in the re-attempters group than single attempters. The items in this K-FAF subscale address self-reproaches, resentments, and mistrust, but also depressive mood (including suicidal ideation). These factors might enhance the probability of suicidal behavior as an extreme type of self-aggressive behavior.

The General Aggression Model (54) provides an excellent theoretical basis to integrate and interpret present results. According to this model (54), three critical stages are consisting of (1) person and situation inputs, followed by (2) a specific internal state including cognition, arousal, affect, and brain activity and (3) appraisal and decision-making processes that could lead to impulsive/aggressive vs. thoughtful actions. Feedback loops can influence future cycles of aggression in terms of learning processes and, thus, enhance the probability of repeating the (self-)aggressive behavior. Accordingly, the repetition of suicide attempts could establish self-aggressive behavior as an automatic (Pavlovian) response (55) to the critical person (e.g., trait anger as an internal state) and situation related factor, e.g., social stress/rejection. In the present study re-attempters experienced significantly more frequently acute stressful events as triggering factors than single attempters, which was also reported by other studies (33, 34). The appraisal of internal states and subsequent decision-making processes (3) are biased by the suicidal mode (56) that is characterized by self-aggressive thoughts (e.g., “I am worthless.”), dysphoria (e.g., anger, sadness, and shame) and physiological arousal, leading to suicidal behavior. In a previous study, self-aggressiveness was related to deficits in emotion regulation in terms of higher impulsivity, low self-directedness, more potent inhibition of aggression, and a tendency to inwardly directed anger (8).

Moreover, we found higher diathesis in suicide re-attempters, i.e., higher vulnerability for suicidal behavior (23). These patients had potentially higher genetic predisposition in terms of significantly more first-degree relatives with suicidal behavior than single attempters and reported more frequently traumatic childhood experiences, which might be associated with specific epigenetic changes (57). Furthermore, the above-discussed personality traits, i.e., aggression, anger, pessimism/hopelessness are known to enhance the vulnerability for suicidal behavior (58). Previous studies showed a significant association between certain aspects of childhood trauma (sexual and emotional abuse, physical neglect) and suicide attempt (10). The epigenetic changes induced by childhood trauma affect gene expression related to HPA axis functioning (59), leading to an abnormal HPA axis activity to stress (60). Furthermore, the experience of childhood trauma was shown to increase impulsivity, diminishing the brain's capacity to inhibit negative actions and control emotions (61). A significant association between childhood trauma and aggressive traits was also observed in several studies (62). Thus, our results indicate, that besides the above-discussed learning processes (as a copy strategy) that could enhance the probability of repeating the (self-)aggressive behavior, the diathesis for suicidal behavior seems to be significantly elevated in the re-attempters compared to the single attempters. This diathesis might lead to a more frequent experience of stress and extremely negative emotions finally leading to suicidal re-attempts or suicide, because of deficits in emotion regulation and of specific favoring personality traits.

Finally, the present findings might also have relevance for designing psychopharmacological, psychotherapeutic and psychosocial interventions. Due to the apparently different risk profiles of single attempters and the re-attempters, it is conceivable to design different intervention approaches for single attempters vs. re-attempters, specifically targeting the more severe psychopathology, deficits in emotion regulation, and problem-solving abilities, higher self-aggressiveness and higher suicidal ideation in re-attempters. As observed in our recent systematic review about psychotherapeutic interventions to prevent suicide re-attempts (63), previous studies applying PT interventions included a varying proportion of multiple5 attempters. However, they did not systematically investigate differences in the PT outcomes between single attempters and re-attempters.

This study must be considered in light of its strengths but also limitations. Data were collected in a cross-sectional design, i.e., few weeks after the suicide attempt. Thus, we cannot interpret our results in a predictive way and do not know about the dynamics/stability of the measured constructs. Furthermore, there might be a selection bias because of our exclusion criteria (acute psychosis, acute intoxication or withdrawal symptoms, diagnosed intelligence impairment, language barriers, lack of insight, and dementia diseases), reducing the generalizability of our results. Additionally, since participation was voluntary, our sample was potentially biased by patients that were more open to talking about their suicide attempt. On the other side, patients that were not willing to talk about their suicide attempt might be a group at higher risk regarding future attempts, potentially showing a more negative reaction toward their survival. Furthermore, we had no prospective information on whether some of the included single attempters will attempt suicide again (33). Finally, the re-attempters might also represent a heterogeneous group, e.g., depending on the number of attempts and the time passed between the attempts. The diathesis might be much more established in re-attempters with a high number of attempts, but also the postulated predominance of automatic (Pavlovian) self-destructive processes (55), which should be considered in tailored treatment strategies.

## Data Availability Statement

The original contributions presented in the study are included in the article/supplementary material, further inquiries can be directed to the corresponding author/s.

## Ethics Statement

This study involving human participants was reviewed and approved by Friedrich-Schiller University, Jena and State Chamber of Physicians of Thuringia, Germany. The patients/participants provided their written informed consent to participate in this study.

## Author Contributions

GW, TS, K-JB, UP, UK, and MW contributed to conception, design and conducting of the study. ML, LB, SJ, AS, and JB organized the database and performed clinical assessments. GW, ML, and JB performed the statistical analysis. ML and GW wrote the manuscript. All authors contributed to manuscript revision, read, and approved the submitted version.

## Funding

This work was supported by a research grant from the Bundesministerium für Gesundheit (BMG; Federal Ministry of Health, ZMVI1-2517FSB143).

## Conflict of Interest

The authors declare that the research was conducted in the absence of any commercial or financial relationships that could be construed as a potential conflict of interest.

## Publisher's Note

All claims expressed in this article are solely those of the authors and do not necessarily represent those of their affiliated organizations, or those of the publisher, the editors and the reviewers. Any product that may be evaluated in this article, or claim that may be made by its manufacturer, is not guaranteed or endorsed by the publisher.
